# Approach or Avoidance? The Mechanisms Underlying the Impact of Community Goal Orientation on Residents’ Waste Separation Recycling Behaviors

**DOI:** 10.3390/bs15010023

**Published:** 2024-12-30

**Authors:** Zhihao Wang, Lingchao Huang, Wei Li, Duo Xu

**Affiliations:** 1 School of Economics and Management, Taiyuan University of Technology, Taiyuan 030024, China; wangzhihao0165@link.tyut.edu.cn; 2 Geshan Holding Group, Dongyang 322100, China; huanglingchao1994@163.com; 3 State Key Joint Laboratory of Environmental Simulation and Pollution Control, School of Environment, Beijing Normal University, Beijing 100875, China; xuduo8025@mail.bnu.edu.cn

**Keywords:** community goal orientation, waste separation recycling behavior, environmental passion, longitudinal study

## Abstract

Amidst the vigorous pursuit of sustainable development, the significance of community management has become increasingly evident. This study, anchored in goal orientation theory, delineated a conceptual model that elucidated the influence of community goal orientation on residents’ waste separation recycling behaviors. Utilizing a longitudinal study design combined with self-report methods, data comprising 871 paired observations from 166 residents were collected and analyzed via multilevel structural equation modeling to test the proposed hypotheses. The findings corroborated that the community approach goal orientation not only had a direct positive impact on the residents’ waste separation and recycling behaviors but could also induce the residents’ environmental passion and subsequently influence their waste separation and recycling behaviors. Conversely, community avoidance goal orientation exhibited no significant effect on either the environmental passion or waste separation recycling behaviors. Furthermore, the community trust was found to positively moderate the effect of community approach goal orientation on the environmental passion. Meanwhile, the community trust moderated the mediating role of environmental passion. However, the community trust did not moderate the effect of the community avoidance goal orientation on environmental passion.

## 1. Introduction

Communities are instrumental in the effective orchestration of resources, services, and communications within the realms of health initiatives and crisis responses (Z. Li et al., 2024; Satsanasupint et al., 2022). In an epoch characterized by rapid urbanization and digital transformation, the significance of community management in advocating for and implementing sustainable development practices has markedly escalated (Gomes et al., 2023; Karres et al., 2022; Y. Zhao & Wise, 2019). In Germany, Spain, Indonesia, and other countries, community management is considered an essential measure (Azevedo et al., 2021; Knickmeyer, 2020; Kubota et al., 2020). In Southeast Asian nations, such as Indonesia, the initiative of “Community Trash Banks” has adeptly harnessed the functional capacity of neighborhood associations, realizing noteworthy outcomes (Kubota et al., 2020). In China, community management initiatives have demonstrated positive outcomes; however, certain communities have implemented fixed schedules for waste disposal to streamline management processes, which has inadvertently resulted in illegal dumping behaviors among residents. Notably, since 2019, X. Wang and Zeng (2024) have categorized community participation into “strong participation” and “weak participation”, revealing that the influence coefficient of community engagement on residents’ waste sorting behavior is merely 0.090. It is clear that waste separation and recycling management, pivotal aspects of sustainability efforts, have increasingly captivated academic focus (W. Li et al., 2024; Soesilo & Alfarizi, 2024; Tan et al., 2024b). Particularly against the backdrop of China’s enforcement of compulsory waste separation policies, the challenge of ascertaining methods through which community management can efficaciously influence residents to refine their waste separation recycling practices emerges as a pressing issue warranting immediate attention.

Previous studies elucidated that communities are pivotal in orchestrating, propagating, and providing guidance; overseeing activities; and managing and maintaining infrastructural facilities. In the realm of oversight, with the extensive implementation of source-separated waste management, communities are advised to prioritize manual surveillance, as well as mobilize volunteers to monitor residents’ behaviors regarding waste segregation at predetermined times and venues (Zelenika et al., 2018). Communities can strategically situate waste segregation and collection points to minimize obstacles for resident participation, thus fostering the convenience of resident engagement in waste-sorting initiatives (Y. Zhang et al., 2022). Nevertheless, the interventions enacted by communities, including oversight mechanisms, are beset by challenges such as significant financial outlays and marginal societal returns (Z. Zhang & Wang, 2020). To minimize expenditures, numerous communities have refined the visual configurations of recycling infrastructures, incorporating distinctive colors, geometries, illustrative directives, and linguistic schematics (Knickmeyer, 2020; Miafodzyeva et al., 2013). Ultimately, communities augment residents’ perception of value and dedication to the communal ethos, cultivating an ambiance of universal participation within the neighborhood, which is instrumental in steering residents toward conscientious and voluntary engagement in waste separation (Pei, 2019).

It is evident that the academic community widely acknowledges the significant role of community management in guiding resident waste separation recycling behaviors. As research progresses, scholars have delved into the mechanisms underlying the influence of community guidance on residents’ waste separation recycling practices. Scholarly inquiries predominantly scrutinized the influence of rational cognitive determinants, including attitudes (Hu et al., 2024), subjective norms (Arkorful et al., 2023; Hu et al., 2021), perceived behavioral control (Kaffashi & Shamsudin, 2019; Ma et al., 2018), moral norms (Khan et al., 2019; Razali et al., 2020), environmental knowledge (S. Wang et al., 2020), perceived cost (Z. Wang et al., 2024b), and recycling habits (Y. Zhang et al., 2022; Aboelmaged, 2021), whilst significantly neglecting the criticality of emotion. Notably, Kanchanapibul et al. demonstrated that emotional factors play a more significant role in fostering pro-environmental behaviors among residents compared with cognitive factors (Kanchanapibul et al., 2014).

It is apparent that specific limitations exist within the current body of research. First, when examining community management and residents’ waste sorting and recycling behaviors, existing studies predominantly focused on rational factors influencing these behaviors while overlooking the significance of emotional factors. Second, the prevailing methodology in current research tends to be cross-sectional, thereby neglecting longitudinal analyses of waste separation and recycling practices. To address these gaps, this study introduced environmental passion as an emotional factor, primarily because it is characterized as a fervent positive emotion and possesses the capacity to propel individuals toward undertaking actions conducive to environmental welfare (Datu & Buenconsejo, 2021; Robertson & Barling, 2013), with its impact frequently surpassing those of cognitive determinants (Kanchanapibul et al., 2014). On the one hand, environmental passion constitutes an experience of profound emotional engagement in environmental preservation, an experience that significantly bolsters an individual’s sense of environmental self-identity (Yin et al., 2021; Z. Wang & Li, 2024). On the other hand, as a positive moral emotion, environmental passion can affect the implementation of pro-environmental behaviors (Shah et al., 2023). Furthermore, this study employed a longitudinal approach to collect data from residents. This was due to the fact that emotional states fluctuate throughout participation in such activities, making cross-sectional datasets inadequate for capturing these temporal dynamics (Gao & Tian, 2019). Conversely, a longitudinal study collects dynamic data at multiple time points, thereby addressing the shortcomings of cross-sectional studies (L. Zhou et al., 2021).

In summary, this study, situated within the context of China’s mandatory waste separation and recycling policy and adopting a community goal orientation perspective, employed a longitudinal approach to investigate the impact of community goal orientation on residents’ waste separation and recycling behaviors. Furthermore, we examined the mediating role of environmental passion in these relationships. Considering the heterogeneity between different communities, we incorporated community trust as a moderating variable to further unveil the mechanisms and boundary conditions of the influence of community goal orientation on resident waste separation behavior.

## 2. Theoretical Foundation and Hypothesis Testing

### 2.1. Goal Orientation Theory

Goal orientation theory epitomizes a comprehensive inclination toward achievement behaviors, steering individuals in their interpretation of events and fostering the genesis of cognitive, emotional, and behavioral paradigms. Diverse goal orientation strategies influence the magnitude of effort individuals exert toward goal-related activities; their selection of goals or tasks; pursuit thereof; and ultimately, task performance. As the theory has evolved, its scholarly exploration has progressively ascended to more elevated planes, with a burgeoning contingent of scholars dedicating attention to the role of goal orientation within group or team dynamics. At the team level, goal orientation signifies a state emergent from its collective perception as an integral component of the team atmosphere (Urdan & Kaplan, 2020). Team goal orientation, as an intrinsic team characteristic, can shape team intentions, which sequentially influence team actions, culminating in outcomes oriented toward the team’s objectives. Previous studies elucidated that team-level goal orientation exerts a significant influence on facets such as information exchange within teams (Gong et al., 2013), creativity in team settings (H. Zhao et al., 2021), overall work performance(Lin et al., 2022), and pro-environmental conduct among teams (Peng et al., 2021).

**Figure 1:**
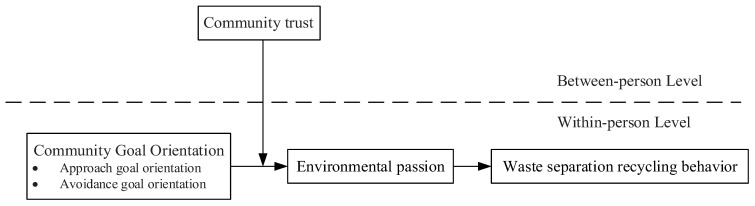
Research model.

Dweck (2017) delineated learning and performance goals within the framework of achievement goal theory. Individuals prioritizing learning goals dedicate their focus to comprehending tasks and augmenting their competencies. Conversely, those prioritizing performance goals harbor aspirations to showcase their intellect and skills to others, albeit shunning scenarios that potentially culminate in failure or reveal their inadequacies. Considering the outcome-centric orientation of waste segregation and recycling initiatives instituted under compulsory policy regimes, which emphasize external assessments over learning processes, this study adopted only the community performance goal. Scholars, notably Weissman and Elliot (2023), further segmented performance goals into performance-approach and performance-avoidance goals. In the pursuit of performance-approach goals, individuals or collectives frequently aspire to garner positive appraisals from peers or organizations during task engagement. Conversely, when aiming for performance-avoidance goals, they generally strive to eschew negative assessments stemming from task failures. Consequently, goal orientation theory provides certain insights into explaining the influence of community goal orientation on waste separation recycling behavior. Based on this, the research framework for the influence of community goal orientation on waste separation recycling behavior is constructed, as shown in Figure 1.

### 2.2. Community Goal Orientation and Waste Separation Recycling Behavior

Within the scope of this research, a community is delineated as a distinct geographic locale inhabited by individuals possessing interconnected relationships and shared attributes (Chen & Yu, 2022). Every member within the community bears the responsibility to engage in waste segregation efforts, given that all individuals produce waste and stand to be directly or indirectly impacted by suboptimal waste management practices (Tan et al., 2024a). Against the backdrop of compulsory policies, pertinent governmental bodies have instituted comprehensive evaluation protocols to gauge the efficacy of domestic waste separation, which involve conducting periodic assessments of community-level waste separation outcomes (W. Li et al., 2024). Communities frequently exhibit divergent objectives embodying distinct goal orientations at the communal level (namely, performance-approach, and performance-avoidance orientation).

Communities characterized by a performance-approach goal orientation endeavor to surpass foundational mandates in waste separation outcomes, aspiring to secure external rewards and garner affirmative assessments from governmental entities, proprietors, and societal sectors (Gong et al., 2013). Communities possessing clear goal delineations, whose behaviors are driven by a performance-approach orientation, underscore the primacy of waste separation. They motivate residents to proactively acquire pertinent knowledge and facilitate intra-community information exchange while aiming for exemplary waste separation achievements (Zheng et al., 2020). The empirical findings of Caniëls et al. (2019) corroborate that within project environments, the team ambience engendered by the performance-approach achievement model significantly contributes to facilitating high-caliber performances among project teams. Ultimately, these communities emphasize impression management, thereby adopting pertinent impression management strategies to cultivate a positive communal image, thereby galvanizing the entire community to dedicate increased efforts toward augmenting the task efficacy.

Communities characterized by a performance-avoidance goal orientation limit their endeavors to fulfilling the minimal stipulations mandated by governmental bodies (Lin et al., 2022). They exhibit a reticence toward dedicating substantial efforts toward the advocacy of waste separation’s significance and its pertinent knowledge, as well as a disinclination to initiate public welfare activities pertaining to waste segregation. Such an approach not only impedes residents’ acquisition of knowledge regarding waste segregation but also obstructs the cultivation of an all-encompassing participatory communal ambience. Within this milieu, residents lacking prior waste segregation experiences are inclined to minimize classification errors, engaging in indiscriminate waste disposal during unsupervised moments, thereby engendering “free-riding” behaviors. Huang et al. evidenced through empirical investigation that an orientation toward failure avoidance within teams indeed exerts a suppressive influence on the collective performance (Huang et al., 2019).

Building upon the aforementioned analysis, this study posited the following hypotheses:

**H1a:**  *Community approach goal orientation positively influences residents’ waste separation recycling behavior.*

**H1b:**  *Community avoidance goal orientation negatively influences residents’ waste separation recycling behavior.*

### 2.3. Mediating Role of Environmental Passion

Passion embodies the fervent predisposition individuals exhibit toward activities they like and deem significant (Yin et al., 2021). Within the realm of green behavior, environmental passion is delineated as a dynamic, positive emotional state driven by an orientation toward environmental preservation (Robertson & Barling, 2013), propelling individuals to contribute positively toward environmental conservation. Existing research indicates that environmental passion plays a crucial role in the relationship between green human resources and employee green behavior (Gilal et al., 2019; Liu et al., 2024). Transformative leadership, green servant leadership, creative leadership, and spiritual leadership can stimulate employees’ environmental passion, thereby enhancing their green behaviors (Afridi et al., 2023; Peng et al., 2021). Corporate social responsibility, by influencing employees’ environmental passion, fosters a green corporate image and promotes eco-friendly behaviors among employees (Ali et al., 2023; Yin et al., 2021). In waste separation initiatives, residents’ environmental passion may lead to heightened awareness of environmental issues and the potential consequences of improper waste management (Z. Wang & Li, 2024). This passion may subsequently drive active participation in waste separation activities; it also motivates residents to exert additional efforts in waste classification, maintaining their commitment, even in the face of inconvenience or a lack of strict enforcement. Additionally, environmental passion is often intertwined with an emotional connection to nature. This emotional bond can further encourage residents to dedicate themselves to waste separation endeavors (Olivos & Clayton, 2017). Consequently, we posit that environmental passion can motivate residents to actively participate in waste separation activities.

Communities focused on performance-approach goals collectively strive for favorable external appraisals. Through organizing activities such as knowledge quizzes, these communities galvanize residents to gather, share information relevant to their tasks, enrich their understanding of waste classification, and enhance their sense of community belonging. This fosters a heightened environmental passion among them (Ali et al., 2023; Procentese & Gatti, 2022). Furthermore, such communities often establish reward and recognition mechanisms to stimulate residents’ enthusiasm and elevate their environmental passion. Lastly, residents of communities with a performance-approach orientation prioritize collective benefits, often engaging in a degree of competition with other communities. The community clearly communicates specific goals and expected achievements of waste separation initiatives to residents, aiding in their understanding of the objectives to be met, thereby sparking their passion for participating in waste classification.

Conversely, communities that pursue performance-avoidance goals are primarily concerned with negative evaluations from organizations or society when their waste separation performance is suboptimal. Under this goal orientation, residents often experience increased external pressure, which may diminish their enthusiasm. Such communities typically lack positive incentives or rewards, relying instead on punitive measures or negative consequences, potentially leading to resident demoralization or resistance, and thus, dampening their environmental fervor (Wu et al., 2021). Finally, these communities often suffer from a lack of adequate and effective waste classification promotion and knowledge-based activities, failing to cultivate a positive community atmosphere. This significantly reduces the interactions between residents regarding waste separation, potentially intensifying their perception of its difficulty and lessening their environmental zeal. Building upon the aforementioned analysis, this study posited the following hypotheses:

**H2a:**  *Residents’ environmental passions play a mediating role in the relationship between community approach goal orientation and residents’ waste separation recycling behavior.*

**H2b:**  *Residents’ environmental passions play a mediating role in the relationship between community avoidance goal orientation and residents’ waste separation recycling behavior.*

### 2.4. The Moderating Role of Community Trust

Community trust encapsulates the subjective assessment by community members regarding the anticipated likelihood of fellow members undertaking specific actions in the future. This evaluative process significantly impacts the propensity of community members to place trust in others or depend upon others’ recommendations in their decision-making processes (Atshan et al., 2020). Especially for members who have lived in a community for a long time, the interactions and connections they establish often influence their behavior judgments (Harring et al., 2019; Hua et al., 2021). Under different community trust atmospheres, the environmental passion exhibited by members based on different goal orientations toward achieving set goals can vary.

When levels of community trust are high, members perceive each other as trustworthy, where they are often filled with positive expectations and a pronounced propensity for reciprocal care (Kohlbacher et al., 2015). This predisposition facilitates a willingness among members to share their classification knowledge and skills, assisting those who have not yet acquired such knowledge. Within this harmonious atmosphere, residents’ environmental passion is easily transmitted to others. In terms of collective interests, elevated levels of trust within the community can effectively mitigate the negative impact of “free-riding” phenomena on residents’ environmental passion (Tam & Chan, 2018). Conversely, in the context of low community trust levels, a deficiency in mutual trust between members significantly erodes the quality of neighborly relations, making it challenging to foster a culture of positive reciprocity within the community. This situation leads to more negative interactions and emotional exchanges between members, thereby diminishing their willingness to participate in waste separation. Additionally, the psychological safety among community residents is compromised (Wickes et al., 2017), accompanied by heightened defensive motivations and intensified fears of losing face. Such dynamics often lead to a preference for silence as a behavioral strategy, suppressing residents’ environmental passion.

In communities characterized by a performance-approach goal orientation coupled with high levels of trust, members engaged in community activities exhibit a greater inclination toward collaboration and mutual support. This fosters a vibrant atmosphere of community interaction, safeguarding collective interests (Liao & Xing, 2023), thereby facilitating the emergence and dissemination of environmental passion. In communities with a performance-avoidance goal orientation and high trust levels, members are predisposed to share pertinent information based on strong interpersonal relationships, even in the absence of specific activities. This dynamic is conducive to alleviating residents’ anxiety and resistance.

Based on the aforementioned analysis, this study proposed the following hypotheses:

**H3a:**  *Community trust positively moderates the relationship between a community approach goal orientation and residents’ environmental passions.*

**H3b:**  *Community trust negatively moderates the relationship between a community avoidance goal orientation and residents’ environmental passions.*

## 3. Method

### 3.1. Participants and Procedures

On 1 July 2019, Shanghai China officially implemented the “Shanghai Municipal Domestic Waste Management Regulations”, heralding the advent of a mandatory era for waste segregation, which has played a significant pioneering role. To deepen the sustainable and effective mechanism of domestic waste segregation and continuously consolidate and enhance its efficacy, Shanghai has commenced regular assessments of the waste segregation status within its communities. Consequently, we conducted a questionnaire survey among the residents of Shanghai. During the survey process, we first communicated with the leaders of the communities under investigation to clarify the objectives and procedures of this study. Following the approval of the community leaders, we randomly selected a segment of residents based on their home addresses to participate in the questionnaire survey. The procedure was as follows: Initially, residents completed a questionnaire that encompassed community trust and demographic variables. Subsequently, over the next eight weeks, residents were tasked with completing a questionnaire every Saturday evening from 8 to 10 PM that covered aspects such as community goal orientation, environmental passion, and waste separation recycling behaviors.

This investigation spanned approximately two months, during which 297 residents completed questionnaires that incorporated measures of community trust and control variables. After the exclusion of 42 questionnaires due to non-serious responses, the effective sample size was determined to be 255. In the subsequent follow-up surveys, a total of 1264 questionnaires were collected. However, due to instances of lost questionnaires or residents failing to complete them on time, 227 were deemed invalid, which resulted in an effectiveness rate of 82.04%. Of these, 89 residents who did not participate in at least three surveys were excluded from our analysis. Retaining data from individuals who completed a minimum of three surveys was deemed essential for capturing changes in the psychological cognition and behavioral patterns among the residents (Gabriel et al., 2018; Z. Wang et al., 2024a). Among the remaining 166 residents, females constituted 56.9%. The majority’s monthly income ranged between RMB 5001 and 20,000, which accounted for 66.7%; age-wise, a significant portion fell within the 26–45 age bracket, which represented 70.5%; and educationally, a substantial number had achieved junior college or undergraduate degrees, which comprised 72.3%.

To facilitate a lagged analysis, questionnaires from week (t) for each participant were aligned with those from week (t + 1), thereby evaluating whether the community goal orientation and environmental passion at week (t) exerted an influence on the waste separation recycling behaviors of residents in week (t + 1). Ultimately, we obtained 871 valid paired data entries from 166 residents. On average, each resident contributed 5.27 paired data entries. The sample size in this study met the criteria set forth by Ohly et al. (2010), which conformed to the between-level of a minimum of 50 samples and a tracking period of at least 5 days.

### 3.2. Measures

Community goal orientation: This study used the scale developed by Gong et al. (2013) to measure the residents’ community goal orientation, where it was adapted to the context of waste classification, which resulted in an 8-item questionnaire. The performance-approach goal orientation included 4 items, such as “Our community is focused on proving that we perform better in waste separation activities than other communities”. The scale had an acceptable reliability (Cronbach’s α = 0.88). The performance-avoidance goal orientation also comprised 4 items, for instance, “Our community is more concerned about negative evaluations for poor waste separation performance than receiving praise or rewards”. The scale had an acceptable reliability (Cronbach’s α = 0.91).

Environmental passion: This study used the scale developed by Robertson and Barling (2013) to measure residents’ environmental passion. The scale contains 10 items, such as “I find pleasure in protecting the environment”. The scale had an acceptable reliability (Cronbach’s α = 0.94).

Waste separation recycling behavior: This study used the scale developed by Razali et al. (2020) to measure residents’ waste separation behaviors. Adapted to fit the Chinese cultural context, the scale was revised, which resulted in 4 items. For example, I frequently separate my household waste. The scale had an acceptable reliability (Cronbach’s α = 0.91).

Community trust: This study used the scale developed by Liao and Xing (2023) to measure community trust. The scale contains 4 items, such as “Residents in our community are trustworthy and reliable”. The scale had an acceptable reliability (Cronbach’s α = 0.92).

This study used a Likert 5-point scale for measuring the variables, with ratings of “Strongly disagree” = 1, “disagree” = 2, “Neutral” = 3, “Agree” = 4, “Strongly agree” = 5.

Control variables: We included gender, age, monthly income level, and educational level as individual-level control variables. Previous studies indicated significant impacts of these factors on the willingness to engage in waste separation behavior (Babaei et al., 2015; W. Li et al., 2020).

### 3.3. Analysis

This study conducted repeated measurements of community goal orientation, environmental passion, and waste separation recycling behavior at the within-person level. At the between-person level, this study measured community trust and control variables (gender, age, educational background, and monthly income). To ensure that within-person level results were not confounded by between-person variance, this study centered the within-person level (Level 1) independent variable (community goal orientation) using each individual’s mean (group mean), which eliminated the impact of the between-person variance. To test the cross-level moderating effect of the between-person level (Level 2) moderating variable (community trust) on the within-person level (Level 1) model, this study centered community trust using the grand mean. During the analysis, we specified the random effects of the independent variable (community goal orientation) on the mediating variables (environmental passion), and the fixed effects of the mediating variables on the dependent variable (waste separation recycling behavior).

Null model tests were conducted with environmental passion and waste separation recycling behavior as the dependent variables, respectively. The results of the null models revealed that the ICC (1) values for each variable were 0.28 and 0.21, both of which exceed 0.05, indicating substantial within-person variability. Consequently, multilevel structural equation modeling (MSEM) was suitable for testing the hypotheses of this study. This study employed Mplus8.3 software to conduct a 1-1-1 path analysis within the framework of MSEM to test the research hypotheses and utilized the Monte Carlo method in R 3.6.3 software to calculate the 95% confidence intervals for the mediation effects (Preacher & Selig, 2012). Lastly, the moderated mediation models were examined using the path analysis method proposed by Sardeshmukh and Vandenberg (2017).

To assess and confirm the discriminant validity of each variable, we conducted a multilevel confirmatory factor analysis (MCFA). In the first model, the community goal orientation, environmental passion, and waste separation recycling behavior were positioned as independent factors at Level 1, while the community trust was selected as an independent factor at Level 2. The results indicate a good fit of the data (χ^2^(205) = 697.43, RMSEA = 0.05, CFI = 0.97, TLI = 0.96, SRMR_within_ = 0.02, SRMR_between_ = 0.01). In the second model, we combined the community goal orientation, environmental passion, and waste separation recycling behavior into one factor positioned at Level 1. However, the results show a poorer fit of the data in this model (χ^2^(211) = 7269.62, RMSEA = 0.20, CFI = 0.53, TLI = 0.47, SRMR_within_ = 0.18, SRMR_between_ = 0.01). Furthermore, it should be noted that the first model’s fit was significantly better than the second model’s fit (Δχ_2_ = 6572.19, Δdf = 6, *p* < 0.01).

## 4. Hypothesis Testing and Results

The means, standard deviations, and correlation coefficients of all the variables are presented in Table 1. At the within-person level, a significant positive correlation was observed between the community approach goal orientation and both the environmental passion (r = 0.35, *p* < 0.01) and the waste separation recycling behavior (r = 0.36, *p* < 0.01). The community avoidance goal orientation was found to be significantly negatively correlated with both the environmental passion (r = −0.11, *p* < 0.01) and the waste separation recycling behavior (r = −0.18, *p* < 0.01). The environmental passion (r = 0.35, *p* < 0.01) was significantly positively correlated with the waste separation recycling behavior. These results aligned with the theoretical expectations and provided preliminary support for the research hypotheses.

The correlations below the diagonal indicate the between-person correlations (*n* = 166), while the correlations above the diagonal represent the within-person correlations (*n* = 871). The diagonal displays the Cronbach’s α coefficients for each variable.

Key—gender: 0 = male, 1 = female; income: 0 = below 5000, 1 = 5001–10,000, 2 = 10,001–15,000, 3 = 15,001–20,000, 4 = above 20,001; education: 0 = high school or below, 1 = junior college degree, 2 = bachelor’s degree, 3 = master’s degree or above.

The path coefficients between all variables are delineated in Table 2. The analysis of Model 2 revealed that the community approach goal orientation had a significant positive effect on the residents’ waste separation recycling behaviors (b = 0.34, *p* < 0.01), thereby corroborating H1a. Conversely, the community avoidance goal orientation did not significantly influence the residents’ waste separation recycling behaviors (b = −0.04, *p* > 0.05), which resulted in H1b not being supported.

The analysis derived from Model 1 illustrated that the community approach goal orientation significantly positively impacted the residents’ environmental passion (b = 0.32, *p* < 0.01), whereas the community avoidance goal orientation did not have a significant influence on the residents’ environmental passion (b = −0.02, *p* > 0.05). Additionally, Model 2 indicated that the environmental passion significantly positively affected the residents’ waste separation recycling behaviors (b = 0.37, *p* < 0.01), suggesting the potential mediation effect of the environmental passion between the community approach and the avoidance goal orientations and residents’ waste separation recycling behavior. This study further employed a Monte Carlo resampling method with 20,000 iterations to test the significance of the mediating effects. The results reveal that the mediating effect of the environmental passion between the community approach goal orientation and the residents’ waste separation recycling behavior was 0.12 with a 95% confidence interval of [0.08, 0.15], not including zero, which supported H2a. Conversely, the mediating effect of environmental passion between the community avoidance goal orientation and the residents’ waste separation recycling behavior was −0.01 with a 95% confidence interval of [−0.03, 0.015], including zero, indicating that environmental passion did not mediate the relationship between the community avoidance goal orientation and the residents’ waste separation recycling behavior, thus H2b was not supported. Moreover, when both the independent variable (community approach goal orientation) and the mediating variable (environmental passion) were regressed on residents’ waste separation recycling behavior in Model 2, the community approach goal orientation still exhibited a significant positive effect, further indicating a partial mediating role of environmental passion in the relationship between the community approach goal orientation and the residents’ waste separation recycling behavior.

**Figure 2:**
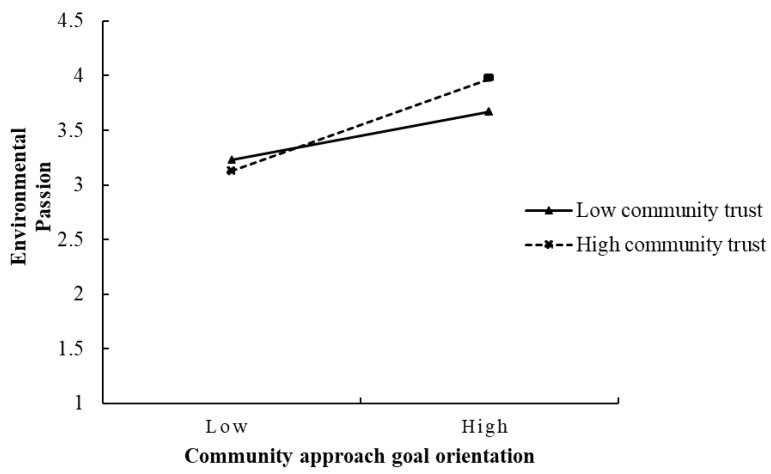
Cross-level moderation effect of community trust on residents’ environmental passion.

The cross-level moderating effect of community trust was examined. The results are presented in Table 2. As indicated by Model 1, the community trust positively influenced the random slope of the community approach goal orientation on the residents’ environmental passion (b = 0.10, *p* < 0.05), which supported H3a. Conversely, the community trust did not significantly impact the random slope of community avoidance goal orientation on the residents’ environmental passion (b = 0.01, *p* > 0.05), indicating that the community trust did not serve as a moderator in the relationship between the avoidance goal orientation and environmental passion, hence H3b was not supported. To clearly illustrate the moderating effects of the community trust, we followed the recommendation of Ozkok et al. (2022) by plotting moderation effect graphs for the community trust varying by one standard deviation (±1SD) (refer to Figure 2). A simple slope analysis of Figure 2 revealed that at the lower levels of community trust (−1SD), the community approach goal orientation significantly positively affected the environmental passion (b = 0.22, *p* < 0.01); at the higher levels of community trust (+1SD), this positive effect became more pronounced (b = 0.41, *p* < 0.01), with a notable difference between the two conditions (b = 0.19, *p* < 0.01), highlighting the moderating influence of the community trust.

We employed the approach proposed by Sardeshmukh and Vandenberg (2017) to examine the moderated mediation model. The data analysis, presented in Table 3, indicates that in the relationship between the community approach goal orientation and the residents’ waste separation recycling behavior, the mediating effect of the environmental passion was 0.15 and significant (95% CI [0.10, 0.21]) under the high community trust. Under the low community trust, this mediating effect was 0.08 and significant (95% CI [0.04, 0.12]), with a significant intergroup difference of 0.07 (95% CI [0.01, 0.13]), demonstrating that the community trust moderated the mediating role of the environmental passion.

## 5. Discussion

Anchored in the innovative exploration of community waste separation recycling management, this study transcended the limitations of previous research that focused on the impact of rational cognitive factors on residents’ waste separation recycling behaviors. It pioneered the investigation of environmental passion as a mediating mechanism, innovatively introducing the influence pathway “Community → Emotion → Behavior”. The theoretical contributions of this research are manifold and can be delineated as follows.

This research substantiated the significant impact of community management on residents’ waste separation recycling behaviors, aligning with prior studies conducted in Japan (Hu et al., 2021), Vietnam(Phan et al., 2023), the United Kingdom (Kolbe, 2019), Singapore (J. Zhou et al., 2022), and South Africa (Thakur & Onwubu, 2024). This consensus across diverse cultural backgrounds and economic levels underscores the indispensable role of community management in the effectiveness of waste segregation initiatives.

Previous studies primarily examined the significance of community management through the lenses of service guidance, rule-making, and facility enhancement (Miafodzyeva et al., 2013; Zelenika et al., 2018). In contrast to the perspectives adopted by earlier scholars, this study approached the issue from the standpoint of community goal orientation, affirming its positive impact on community approach goal orientation. This finding not only aligns with the proposals by W. Li et al. (2024) and Phan et al. (2023) to integrate community management with resident behavior in order to elucidate variations in behavioral intentions among residents but also validates the applicability of goal orientation theory within pro-environmental behaviors, thereby expanding the theoretical framework’s scope.

Previously, Yin et al. (2021) posited that future investigations should delve into a broader spectrum of positive emotions to catalyze residents’ autonomous motivation, thereby fostering voluntary pro-environmental behaviors (Cheng et al., 2023). Specifically, Y. Wang and Hao (2020) emphasized the necessity of implementing more efficacious measures at the community level to engage residents’ intrinsic motivations. In response to the aforementioned recommendations, this study, from a community perspective, introduced environmental passion and explored the mechanism by which community goal orientation influences residents’ waste separation recycling behaviors. Additionally, while previous studies predominantly utilized cross-sectional data to test hypotheses, noting that residents’ emotional levels fluctuate over time (Gao & Tian, 2019), this research employed a longitudinal study, thus filling a methodological gap in the study of waste separation recycling behaviors.

However, this study was not without its limitations. It was confined to the context of China and did not address the applicability of the theoretical model in other countries or across diverse cultures. Moreover, it did not examine whether the mechanisms of action of this theoretical model varied across different cultural contexts. Future research should further investigate whether the impact of community goal orientation on residents’ waste separation recycling behaviors varies across different cultural settings. Prior research demonstrated that factors such as environmental education, outdoor activities, and corporate social responsibility positively influence environmental passion (Junot et al., 2017; Z. Wang & Li, 2024). However, this study focused exclusively on the impact of community goal orientation on residents’ environmental passion, without accounting for other influencing factors.

Second, this study confirmed that community avoidance goal orientation had neither a positive nor a negative impact on residents’ waste separation and recycling behavior. This conclusion contradicts some previous research findings, which may be directly related to the omission of other influencing factors in this study. Additionally, the reliance on residents’ self-reported community goal orientation during the data collection might have contributed to this discrepancy. Future studies should develop more scientific and rigorous methods and incorporate various influencing factors to better explain this contradiction. Moreover, this study solely utilized the self-report method to assess residents’ waste sorting and recycling behaviors. Future research should aim to enhance the measurement techniques to gather more accurate data on these behaviors. Lastly, future investigations are required to delve deeper into the mechanisms through which community goal orientation influences waste separation recycling behaviors, uncovering additional mediating mechanisms and boundary conditions.

## 6. Conclusions

Drawing upon goal orientation theory, this investigation systematically examined the influence of community goal orientations on residents’ waste separation recycling behaviors, alongside the mediating mechanisms and boundary conditions involved. First, the community approach goal orientation had a direct positive impact on the residents’ waste separation recycling behaviors. H1a was supported. The community avoidance goal orientation did not influence the waste separation recycling behaviors among the residents. H1b was not supported. Second, the residents’ environmental passion played a mediating role in the relationship between the community approach goal orientation and the residents’ waste separation recycling behavior. In other words, an orientation toward approach goals within the community was found to foster the residents’ environmental passion, which subsequently enhanced the waste separation recycling behaviors. H2a was supported. In contrast, an orientation toward avoidance goals within the community did not impact the residents’ environmental passion. The residents’ environmental passion did not play a mediating role in the relationship between the community avoidance goal orientation and the residents’ waste separation recycling behavior. H2b was not supported. Third, community trust was demonstrated to moderate the positive influence of an approach-oriented goal within the community on the residents’ environmental passion. H3a was supported. The community trust enhanced the mediating role of the environmental passion in the relationship between the community avoidance goal orientation and the residents’ waste separation and recycling behavior. Nonetheless, the community trust did not moderate the effect of the avoidance goal orientation within the community on the residents’ environmental passion. H3b was not supported.

This study has significant implications for the management practices of waste segregation. Initially, it demonstrated that the community goal orientation substantially exerted a direct positive influence on the residents’ waste separation and recycling behaviors. Supervisory bodies responsible for community management should implement relevant training and promotional activities to help community managers understand the importance of waste segregation and enhance their engagement in these initiatives. The introduction of diversified performance indicators is essential for evaluating not only the tangible outcomes of waste segregation but also the frequency of the related community activities and residents’ satisfaction levels. Additionally, it was recommended to establish a reward mechanism to recognize communities that achieve exceptional results in waste segregation.

Second, considering the mediating role of environmental passion, communities should vigorously conduct promotional and educational activities to strengthen the residents’ awareness of environmental protection and ignite their passion for the environment. Communities are encouraged to involve residents in environmental protection volunteer activities, such as tree planting, to foster environmental passion. Establishing environmental reward mechanisms to honor residents who make significant contributions to environmental protection can motivate more individuals to participate in such activities. Regularly sharing the outcomes of community environmental improvements with residents can enhance their satisfaction and sense of belonging.

Lastly, this study illustrates that community trust can amplify the positive effect of the community’s approach goal orientation on residents’ environmental passion. This serves as a reminder for community managers to place high emphasis on building community trust and atmosphere. For instance, organizing cultural activities and waste segregation practical activities can provide a platform for residents to interact and cooperate. Through these activities, residents can better understand each other, foster mutual trust, and enhance community identification and trust by encouraging resident participation in community decision-making and organizing mutual aid activities, like neighborhood assistance groups and volunteer services, thereby strengthening the trust between community members.

## Figures and Tables

**Table 1:** Correlations.

	M	SD	1	2	3	4	5	6	7	8	9
Between-person level											
1. Sex	1.57	0.50									
2. Age	2.87	0.84	−0.07 *								
3. Income	2.58	0.89	0.04	0.01							
4. Education	2.11	1.32	0.07 *	−0.01	0.15 **						
5. Community trust	3.69	1.08	0.04	0.10 **	0.12 **	−0.01	(0.92)				
Within-person level											
6. Approach goal orientation	3.66	0.52	−0.03	0.14 **	0.04	−0.03	0.10 **	(0.88)	−0.12 **	0.35 **	0.36 **
7. Avoidance goal orientation	2.46	0.62	−0.01	0.07 *	−0.11 **	−0.11 **	0.19 **	−0.06	(0.91)	−0.11 **	−0.18 **
8. Environmental passion	3.20	0.52	0.01	−0.03	0.03	0.02	−0.03	0.38 **	−0.15 **	(0.94)	0.35 **
9. Waste separation recycling behavior	3.50	0.58	0.01	0.06	0.01	0.14 **	−0.39 **	0.34 **	−0.29 **	0.30 **	(0.91)

Note: * *p* < 0.05, ** *p* < 0.01.

**Table 2:** Results of multilevel regression analysis.

Variable	Environmental Passion		Waste Separation Recycling Behavior	
	Model 1		Model 2	
	Estimate	SE	Estimate	SE
Intercept	3.30 **	0.24	3.27 **	0.26
Within-person level				
Approach goal orientation	0.32 **	0.04	0.34 **	0.04
Avoidance goal orientation	−0.02	0.03	−0.04	0.04
Environmental passion			0.37 **	0.04
Between-person level				
Gender	−0.01	0.08	−0.01	0.09
Age	−0.03	0.05	0.04	0.05
Education	0.01	0.05	−0.01	0.05
Income	0.01	0.03	0.06	0.03
Community trust	0.05	0.04		
Cross-level moderation				
Approach goal orientation × community trust	0.10 **	0.03		
Avoidance goal orientation × community trust	0.01	0.03		
Within-level residual variance	0.42 **	0.03	0.57 **	0.03
Between-level residual variance	0.20 **	0.03	0.21 **	0.04
R^2^	0.13		0.23	

Note: ** *p* < 0.01. All results were derived from a model test that included all variables.

**Table 3:** Results of moderated mediating effect.

Mediating Variables	Level of Moderating Variable	Estimate	SE	95%CI
Environmental passion	Low community trust	0.08	0.03	[0.04, 0.12]
	High community trust	0.15	0.02	[0.10, 0.21]
	Difference	0.07	0.03	[0.01, 0.13]

## Data Availability

The data presented in this study are available on request from the corresponding author.
